# Species delineation and systematics of a hemiclonal hybrid complex in Australian freshwaters (Gobiiformes: Gobioidei: Eleotridae: *Hypseleotris*)

**DOI:** 10.1098/rsos.220201

**Published:** 2022-07-27

**Authors:** Christine E. Thacker, Daniel L. Geiger, Peter J. Unmack

**Affiliations:** ^1^ Department of Vertebrate Zoology, Santa Barbara Museum of Natural History, 2559 Puesta del Sol, Santa Barbara, CA 93105, USA; ^2^ Department of Invertebrate Zoology, Santa Barbara Museum of Natural History, 2559 Puesta del Sol, Santa Barbara, CA 93105, USA; ^3^ Research and Collections, Section of Ichthyology, Natural History Museum of Los Angeles County, 900 Exposition Blvd., Los Angeles, CA 90007, USA; ^4^ Centre for Applied Water Science, Institute for Applied Ecology, University of Canberra, Canberra, ACT 2601, Australia

**Keywords:** Eleotridae, *Hypseleotris*, freshwater, hybrid, hemiclone, systematics

## Abstract

The rivers of southeastern Australia host a species complex within the carp gudgeon genus *Hypseleotris* that includes parental species and hemiclonal hybrid lineages. These hemiclones can be difficult to distinguish from their parent taxa, making delineation of species unusually difficult. We approach this historical taxonomic problem by using single nucleotide polymorphism (SNP) genotyping to distinguish individuals of each species and hemiclones, enabling us to quantify the variation among evolutionary lineages and assign names to the species. *Hypseleotris klunzingeri* remains valid and does not have any hemiclones. We describe *Hypseleotris bucephala* and *Hypseleotris gymnocephala* from the Murray-Darling Basin and *Hypseleotris acropinna* from the Murray-Darling as well as eastern coastal streams north of the Mary River, part of the range attributed to *H. galii*. We further split *H. galii* to distinguish a species from the Mary River, *Hypseleotris moolooboolaensis*. We designate a neotype and redescribe *H. galii* due to uncertainty about the source and species identity of specimens used in the original description. We reconcile previous taxonomies, provide new common names for parental species, and advocate using the scientific names of both parents when referring to the hemiclone hybrids to avoid confusion with previous common names that did not distinguish parental taxa and hemiclones.

## 1. Introduction

Traditional methods of animal species identification and delineation rest on the assumption that evolutionary lineages are isolated from one another and do not hybridize (reticulate) with any other lineages. This fundamental premise is embedded in all but the most general species concepts and also serves as a ground assumption when constructing strictly bifurcating phylogenetic trees. However, in cases where lineages reticulate after diverging, species definitions for both the parent and daughter taxa are more difficult to assign. If lineages are interfertile they violate the central tenet of the biological species concept (reproductive isolation), and yet it is sometimes possible for parent lineages to generate a hybrid lineage and for both parents and hybrids to persist as cohesive lineages themselves. These lineages formed from hybridization may reproduce unisexually, a rare occurrence that has been documented in vertebrates including fishes, reptiles and amphibians, along with a diversity of invertebrate groups [1–6]. Three types of unisexual reproduction are known among vertebrates: parthenogenesis, gynogenesis and hybridogenesis [7]. In parthenogenesis, reproduction occurs without any mating and all offspring are identical to their mother. With gynogenesis, mating is required but the sperm only triggers egg development and the male genome is not incorporated into the zygote, so offspring are full clones of the female unisexual parent. In hybridogenesis offspring incorporate, without genetic recombination, the haploid genome of the sexual parent, which is then discarded during gametogenesis and replaced during fertilization in the following generation. Reproduction is therefore ‘hemiclonal’, since the haploid genome derived from the unisexual parent is passed on without modification [8]. Most known hemiclones are all-female, but rare instances of male hemiclones are known, including among hybridogenic lineages of the Australian freshwater fish genus *Hypseleotris* [9].

*Hypseleotris* (carp gudgeons) are small fishes (most species attain adult lengths of 30–50 mm SL), which superficially resemble cyprinids (laterally compressed, with small, upturned mouths) [10,11]. They inhabit fresh and occasionally brackish waters and are generally demersal. *Hypseleotris* are not close relatives of other Australian Eleotridae, and neither morphological nor molecular phylogenetic studies have yielded a confident placement of *Hypseleotris* within the family [12,13]. Among the southeastern species, *H. compressa*, *H. galii* and *H. klunzingeri* are the only taxa that have been formally described [14]. Three additional taxa have been informally recognized [15]: Midgley's carp gudgeon, Lake's carp gudgeon and the Murray-Darling carp gudgeon [16], a close relative of *H. galii*. With the exceptions of *H. compressa* and *H. klunzingeri*, all the southeastern Australian *Hypseleotris* have hybridized with each other to form multiple co-occurring stable hemiclonal unisexual lineages [9,11,17]. Hemiclonal lineages in *Hypseleotris* are genetically identifiable but morphologically range from intermediate too difficult to distinguish from one of their parental taxa. Without an understanding of the range of genotypes among southeastern Australian *Hypseleotris*, it has been impossible to unravel the morphological variation seen among individuals, correlate morphology with genetics and unambiguously assign taxonomic names [18–21]. Moreover, the three informal taxon names refer to parental species as well as hybridogenic lineages, further complicating the taxonomy. A history and concordance of names for taxa within the *Hypseleotris* hemiclonal species complex is given in table 1. Genetic patterns among *Hypseleotris* species and their hemiclones were first investigated using nuclear markers (allozymes) in the Lower Murray River (South Australia) in the Murray-Darling Basin (MDB) [17]. That study established the presence of three distinct allozyme genotypes (HA, HB, and HX), with HX found in that study only as a part of hybrid combinations with the other genotypes. Those genotypes correspond to *H. galii*/Murray-Darling carp gudgeon, Midgley's carp gudgeon and Lake's carp gudgeon, respectively. An additional allozyme genotype (HC) was assigned to *H. klunzingeri*, a southeastern species that does not form hemiclonal hybrids. In addition to the genetic distinctions, the species and hemiclones were separable in a discriminant function analysis based on scale counts, fin element counts and vertebral number, although the individual meristic counts may overlap [17]. An analysis of *Hypseleotris* from the Goulburn River system in Victoria (part of the MDB) based on cytochrome *b* sequence and microsatellite markers confirmed the presence of the genotypes delineated in [17] and added information on hemiclone sexual combinations and frequency as well as demonstrating that pure species and hemiclonal lineages were distinguishable in cluster and principal component analyses of the genetic data [9]. Most recently, an expanded study with samples from across the MDB (plus adjoining parts of the Lake Eyre Basin (LEB), Bulloo River and coastal South Australia) used phylogenetic analysis of mitochondrial cytochrome *b* as well as nuclear single nucleotide polymorphism (SNP) data to identify all of the parental and hemiclonal lineages in southeastern *Hypseleotris* including a remnant population of the pure parental HX genotype (Lake's carp gudgeon) in a small tributary of the upper Lachlan River (MDB) [22]. In this hemiclonal system, once a hybrid individual is formed, the chromosomal complement of one parent is eliminated from the gametes generated by that individual and only the genes of the other parent are passed (without recombination) to the hemiclonal offspring. The chromosomal mechanisms of this process were elucidated by [23].

**Table 1.** Historic formal and informal nomenclature for *Hypseleotris* species of southeastern Australia and their taxonomic status.

source	nominal taxa/informal names	taxonomic status after this study	note
[14]	*Carassiops* *klunzingeri*, Murray River, South Australia	*Hypseleotris klunzingeri* [14]	
[14]	*Carassiops galii*, Botanical Gardens, Sydney	*Hypseleotris galii* [14] in part	Neotype designated here due to uncertain geographical location of source population
[10,15,16,20]	*Hypseleotris klunzingeri*, southeast Australia	*Hypseleotris klunzingeri* [14]	
[10,15,16,20]	*Hypseleotris galii*, coastal southeast Australia	Northern range = *Hypseleotris acropinna* new species, in part (also MDB); Mary River = *Hypseleotris moolooboolaensis* new species; Southern range = *Hypseleotris galii* [14]	Also encompasses combinations of hemiclones with *Hypseleotris bucephala* new species and *Hypseleotris gymnocephala* new species
[15,20]	Midgley's carp gudgeon (*Hypseleotris* sp. 4)	*Hypseleotris bucephala* new species; *H. acropinna* × *H. bucephala* (HA × HB)	
[15,20]	Lake's carp gudgeon (*Hypseleotris* sp. 5)	*H. acropinna* × *H. gymnocephala* (HA × HX); *H. bucephala* × *H. gymnocephala* (HB × HX)	
[16]	Murray-Darling carp gudgeon (*Hypseleotris* sp. 3)	*Hypseleotris acropinna* new species, *H. acropinna* × *H. gymnocephala* (HA × HX)	
[17]	HC (corresponds to *H. klunzingeri*)	*Hypseleotris klunzingeri*	Not involved in hybridogenic complex
[17]	HA (corresponds to Murray-Darling carp gudgeon in part)	*Hypseleotris acropinna* new species, in part (also coastal)	F1 hybrids with HB and HX (suspected hemiclones)
[17]	HB (corresponds to Midgley's carp gudgeon)	*Hypseleotris bucephala* new species	F1 hybrids with HA and HX (suspected hemiclones)
[17]	HX (ancestral parent of Lake's carp gudgeon, pure fish not found, but see Unmack *et al*. [22])	Hemiclonal lineages involving *Hypseleotris gymnocephala* new species	F1 hybrids with HA and HB (suspected hemiclones); only as hybrid signature
[10]	Midgley's carp gudgeon (*Hypseleotris* species 1)	*Hypseleotris bucephala* new species; *H. acropinna* × *H. bucephala* (HA × HB)	
[10]	Lake's carp gudgeon (*Hypseleotris* species 2)	*H. acropinna* × *H. gymnocephala* (HA × HX); *H. bucephala* × *H. gymnocephala* (HB × HX)	
[10]	Murray-Darling carp gudgeon (*Hypseleotris* species 3)	*Hypseleotris acropinna* new species, in part; *H. acropinna* × *H. gymnocephala* (HA × HX)	
[21]	HA (corresponds to Murray-Darling carp gudgeon)	*Hypseleotris acropinna* new species	
[21]	HA × HB	*H. acropinna* × *H. bucephala*	
[21]	HA × HX	*H. acropinna* × *H. gymnocephala*	
[21]	HB (corresponds to Midgley's carp gudgeon)	*Hypseleotris bucephala* new species	
[21]	HB × HX	*H. bucephala* × *H. gymnocephala*	
[21]	HX (ancestral parent of Lake's carp gudgeon)	*Hypseleotris gymnocephala* new species	

These studies have determined the geographical ranges of various genetic forms within southeastern Australian *Hypseleotris* and enabled assignment of morphotypes to genetic lineages. This allows discrimination of individuals representing pure parental species from their hemiclones, which is necessary in order to formally describe the species and clarify morphological distinctions among species and hemiclones. For this study, we examine representatives of all southeastern Australian *Hypseleotris* species and their hemiclones and confirm identity of all the examined individuals with SNP genotyping. Our aim is to update the taxonomy of the group and to compare pure and hemiclonal populations of known genotypes to evaluate whether and how the pure species and their hemiclones may be distinguished from one another morphologically. We then describe four *Hypseleotris* species, provide a redescription and neotype for *H. galii*, and discuss the use of names (common and scientific) in this group as it relates to their unusual reproductive biology [9,17,22].

## 2. Material and methods

### 2.1. Specimen collection, identification and preservation

We base our descriptions and designate types from a large series of *Hypseleotris* collected throughout Queensland, New South Wales, Victoria and South Australia between 2014 and 2018 that encompass many localities across the MDB, Cooper Creek (LEB) and east coast drainages. We collected fishes using a seine net and euthanized them humanely with an overdose of clove oil. Fishes were collected and processed under University of Canberra animal ethics approvals CEAE 13-06, CEAE 15-06 and 20180442. After collection, we provisionally sorted fishes to species and hybrid hemiclonal lineages in the field. We removed a small piece of muscle from the right side of the caudal peduncle for each fish and preserved it in liquid nitrogen for genotyping, then preserved individual fish in 5 ml tubes in buffered 10% formalin. After a few weeks we transferred the samples to 70% ethanol. First, fish had two soaks in water for approximately 12 h, followed by three changes of 30% ethanol, 50% ethanol and 70% ethanol for 2–12 h each and rinsed with a final replacement of 70% ethanol. A complete list of specimens examined for genotype and morphology, including their specimen codes, identification and collection data is given in electronic supplementary material, table S1.

### 2.2. SNP genotyping

For each individual, we confirmed (or reassigned) the field identifications based on the nuclear SNP genotype, using two similar reference sets based on medium- and low-density SNP coverage. PCA analyses were conducted on the medium-density SNP reference set which included 278 individuals and the low-density set which included 167 individuals, spanning in both cases the taxonomic and geographical diversity of the hemiclonal complex. We performed phylogenetic analyses on the medium-density SNP reference dataset consisting of 240 individuals and the low-density SNP reference dataset consisting of 143 individuals, each including the non-hybrid representatives of the hemiclonal complex plus *Hypseleotris klunzingeri*. We excluded hemiclone hybrids from the phylogenetic analyses because they violate the assumption of non-reticulate ancestry. All genotyped individuals present in the SNP analyses are listed in electronic supplementary material, table S2. Sequencing for the SNP datasets was performed using DArTseq (DArT Pty Ltd, Canberra Australia) or DArTseqLD using medium- and low-density coverage, respectively. DArTseq is a variation of the double-digest RAD technique which combines next-generation sequencing, complexity reduction using restriction enzymes, and implicit fragment size selection [24]. Complexity reduction used the restriction enzymes SbfI (recognition sequence 5′-CCTGCA|GG-3′) and SphI (5′-GCATG|C-3′). All details of the sequencing methods used follow the protocols outlined in [25].

We filtered and error-checked the SNP datasets in two phases, one included automatically as part of DArTseq standard protocols [25], followed by various operator-defined choices implemented on the raw datasets using the R package dartR 2.2.3 [26]. We then filtered the error-checked datasets to remove loci that did not show greater than 99% reproducibility (reproducibility = 0.99) for the approximately 30% of individuals that are routinely randomly re-sequenced by DArT. For the medium-density dataset we removed loci displaying more than 30% missing genotypes (callrate by locus ≥ 0.7). Due to the lower number of available loci in the low-density dataset we retained all loci for the PCA analysis, but to reduce missing data for the phylogenetic analysis, we removed loci displaying more than 50% missing genotypes (callrate by locus ≥ 0.5) in the phylogenetic matrices. To confirm identifications of the specimens examined, we performed PCA on both of the SNP datasets and evaluated the composition of each cluster. Finally, to confirm the monophyly of the parental species, we generated phylogenetic trees for the parental species only (hemiclones removed) for each of the SNP datasets (low and medium-density) using maximum likelihood (ML) applied to concatenated sequences. ML analyses were conducted using RAxML 8.2.12 [27] on the CIPRES cluster [28] using the model GTRCAT and searching for the best-scoring ML tree using the model GTRGAMMA.

### 2.3. Morphological analyses

Based on the combination of field identifications and clustering patterns seen in the SNP PCAs and the ML phylogenies, we selected 255 genotyped individuals for morphological examination, focusing on external characters that can be easily measured and used for identification. For each individual, we counted dorsal, anal and pectoral fin elements; the final dorsal and anal elements were branched to the base and were counted as one element. We also counted the number of lateral scales from the pectoral fin axil to the centre of the caudal peduncle and the number of head scales at the dorsal midline from the base of the first dorsal fin anteriad to the interorbital region. For the pectoral fin and lateral scale counts we performed counts on the left side of the fish only because muscle samples from the caudal peduncle had been removed on the right side of each fish, except in rare cases where the left pectoral fin was damaged and so the right was counted. We present the counts for each species in table 2.

**Table 2.** Morphological (meristic and descriptive) characters for southeastern Australian *Hypseleotris* species examined in this study. All identifications were confirmed by genotyping. The frequency of counts observed is listed in parentheses. Counts of holotypes (or neotype) for *H. acropinna*, *H. bucephala*, *H. galii*, *H. gymnocephala*, and *H. moolooboolaensis* are indicated with asterisks. Dorsal fin spines are denoted with Roman numerals and are counts of elements in the first dorsal fin. Dorsal fin ray counts are counts of the second dorsal fin elements and begin with a Roman numeral I (indicating a spine), followed by the ray count in Arabic numerals. Anal fin ray counts are denoted in the same manner.

species	dorsal fin spines	dorsal fin rays	anal fin rays
*acropinna*	VI(8),VII(38*)	I,10(10),11(34*),12(2)	I,10(1),11(25*),12(18),13(2)
*bucephala*	VI(1)VII(48*),VIII(29)	I,10(11),11(36),12(31*)	I,10(5),11(41),12(32*)
*galii*	VII (12*)	I,11(11*),12(1)	I,11(6*),12(6)
*gymnocephala*	VII(10),VIII(10*)	I,10(1),11(17*),12(2)	I,11(16*),12(4)
*klunzingeri*	VI(6),VII(2)	I,10(5),11(3)	I,10(5),11(3)
*moolooboolaensis*	VII(8),VIII(7*)	I,10(2),11(5), 12(8*)	I,12(15*)
species	pectoral fin rays	longitudinal scales	predorsal scales
*acropinna*	13(3),14(35*),15(8)	31*–34	12*–18
*bucephala*	13(16),14(45),15(17*)	30–35*	0–19 (18*)
*galii*	15(8*),16(4)	31*–33	12–14 (13*)
*gymnocephala*	15(11),16(8*),17(1)	28–35 (32*)	absent
*klunzingeri*	13(1),14(6),15(1)	30–32	12–15
*moolooboolaensis*	15(15*)	33–34*	15–19 (16*)
species	male head shape	vertebrae	male fin band colour & shape
*acropinna*	intermediate	29–30	pale orange, elongated
*bucephala*	blunt	29–30	red, black, orange, white
*galii*	intermediate	29–31	black, red, elongated
*gymnocephala*	blunt	30–31	pale orange, white
*klunzingeri*	pointed	26–29	red, white
*moolooboolaensis*	intermediate	29–30	pale orange, elongated

## 3. Results

### 3.1. SNP genotyping and phylogeny

For the PCA analyses we recovered 28 207 polymorphic SNP loci for 278 *Hypseleotris* individuals in the medium-density SNP dataset and 1823 polymorphic SNP loci for 167 *Hypseleotris* individuals in the low-density SNP dataset. After filtering for repeatability (0.99) and call rate by locus (0.7), the medium-density dataset retained 24 883 and then 13 605 loci and all individuals had a call rate above 58%, with the missing data most likely due to mutations at the enzyme cutting sites rather than being present but not sequenced. The low-density dataset included 1497 loci after filtering for repeatability. One individual had a call rate of 31%, with all remaining samples being over 40%. The PCA plots for *Hypseleotris* species and hemiclones are shown in electronic supplementary material, figure S1.

**Figure 1.**
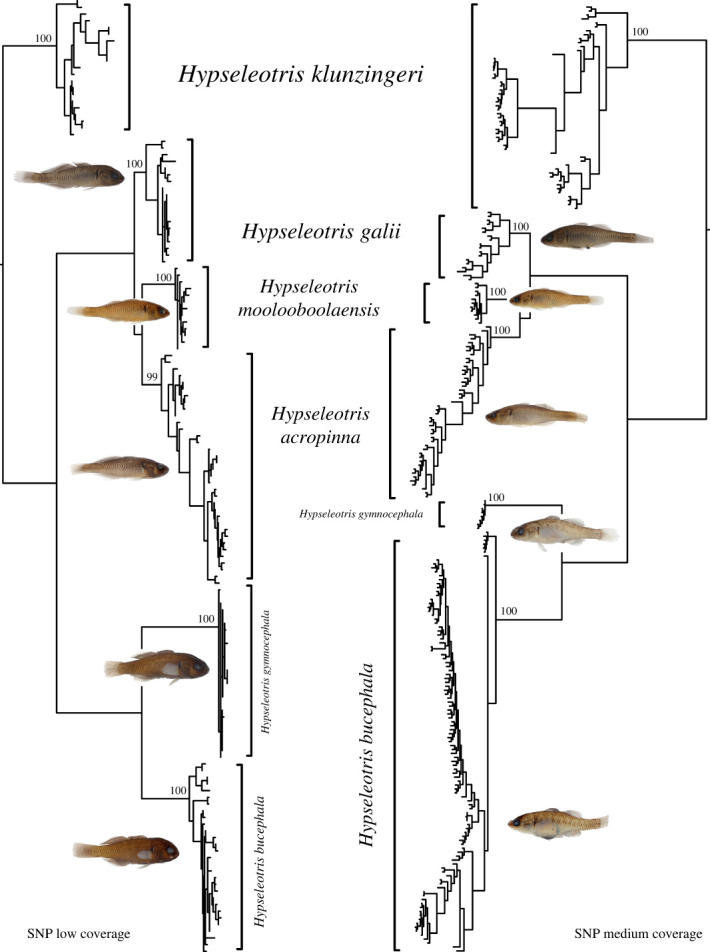
Maximum-likelihood phylogenies of the SNP low-coverage dataset (left) and SNP medium-coverage dataset (right) for genotyped individuals of southeastern *Hypseleotris* species, with photos of males (left) and females (right) shown (not to scale). Numbers at nodes subtending each species are bootstrap support values. Detailed phylogenies of each dataset with tip labels and support values for all nodes are given in electronic supplementary material, figures S2 and S3.

For the phylogenetic analyses we recovered 35 976 polymorphic SNP loci for 240 *Hypseleotris* individuals in the medium-density SNP dataset and 2137 polymorphic SNP loci for 143 individuals in the low-density SNP dataset. After filtering for repeatability (0.99) and call rate by locus (0.7), the medium-density dataset retained 32 630 and then 14 745 loci and all individuals had a call rate above 55%. The low-density dataset included 1805 loci after filtering for repeatability, and 890 loci after filtering for call rate by locus (0.5). Two individuals had a call rate of 33 and 37%, with all remaining samples being over 47%. The concatenated SNP variants for the medium- and low-density datasets are given in electronic supplementary material, tables S3 and S4. Phylogenies of southeastern *Hypseleotris* individuals examined in both the medium- and low-density SNP datasets are given in figure 1, with detailed phylogenetic results for each dataset shown in electronic supplementary material, figures S2 and S3.

Based on the genetic and morphological data, we describe four new *Hypseleotris* species, replacing the three informal names (Lake's carp gudgeon, Midgley's carp gudgeon and Murray-Darling carp gudgeon) and also distinguishing a fourth new species for individuals similar to but distinct from *H. galii* in the Mary River. These species are formally described below, but for clarity, we use the new scientific and common names throughout the rest of the paper: *H. gymnocephala* for the bald carp gudgeon (formerly Lake's carp gudgeon); *H. bucephala* for the boofhead carp gudgeon (formerly Midgley's carp gudgeon); *H. acropinna* for the cryptic carp gudgeon (formerly Murray-Darling carp gudgeon); and *H. moolooboolaensis* for the Mary carp gudgeon.

### 3.2. Morphology and taxonomy

We selected and examined a total of 255 genotyped individuals for morphology: 20 *H. gymnocephala*, 78 *H. bucephala*, 46 *H. acropinna*, 15 *H. moolooboolaensis*, 12 *H. galii*, 29 *H. gymnocephala* × *H. bucephala* hybrids, 26 *H. acropinna* × *H. bucephala* hybrids, 4 *H. moolooboolaensis* × *H. bucephala* hybrids, 3 *H. galii* × *H. bucephala* hybrids, 14 *H. acropinna* × *H. gymnocephala* hybrids and 8 *H. klunzingeri*. We did not examine hybrids between *H. galii* and *H. gymnocephala*, which are rare and may be the result of translocations given their native ranges have never been recorded to overlap. Hybrids between *H. moolooboolaensis* and *H. gymnocephala* have not been recorded, and are similarly unlikely since the species do not co-occur. Using only genotyped individuals for our morphological survey enables us to definitively assign character states to taxa, overcoming the primary difficulty in *Hypseleotris* identifications: uncertainty as to species boundaries. We summarize the characters and meristic counts of the pure *Hypseleotris* species in table 2, and discuss the variation present among the hybrid hemiclones. In both table 2 and in the descriptions below, the number of dorsal fin spines is indicated with Roman numerals and the second dorsal and anal fin elements are denoted as having a single spine (Roman numeral I) followed by the number of fin rays in Arabic numerals.

The species are sexually dimorphic, so for each one we designate a male holotype and female allotype and deposit these specimens at Museums Victoria (NMV). We additionally deposit sets of paratypes (male and female) in the Australian Museum, Sydney (AMS) and Natural History Museum of Los Angeles County (LACM). Separating *H. acropinna* and *H. moolooboolaensis* from *H. galii* restricts *H. galii* to eastern coastal drainages south of the Mary River. Additionally, no holotype was designated for *H. galii* when it was described, so for clarity we provide a redescription of *H. galii* here and select genotyped specimens for designation as neotype (female) and additional voucher (male), also deposited at the AMS.

### 3.3. Systematics

***Hypseleotris* Gill, 1863**

*Hypseleotris* Gill, 1863: 270 (type species: *Eleotris cyprinoides* Valenciennes 1837, by original designation)

Genus of Eleotridae with compressed head, body, mouth small, oblique, not extending beyond middle of eye. Gill opening extending to below posterior end of preoperculum. Pectoral base narrow, with dark pigment blotch on dorsal margin. Pelvic fins separate, each pelvic fin with one spine and five segmented rays. Body scales large, ctenoid; predorsal area (nape) and cheek variably scaled with cycloid scales. Head pores present or absent (absent in all species considered here). Teeth small, monomorphic, in several rows in both jaws. First dorsal VI–IX; dorsal fin insertion pattern 3-1221 or 3-12210; second dorsal I, 10–13; anal I, 10–13, pectoral rays 13–17. Anal fin originating directly below origin of second dorsal fin. Anal fin elements 6–11 preceding the first haemal spine. Vertebrae 25–33. Species considered here sexually dimorphic, with mature males exhibiting heads enlarged in the dorsal/nape region, brightly coloured dorsal anal fins during the breeding season.

This description combines our observations with the most prominent characters given in [29], along with additional characters from [10,11,30].

***Hypseleotris acropinna* Thacker, Geiger & Unmack sp. nov.**

urn:lsid:zoobank.org:pub:7ECCD2C4-302E-447F-BFCA-AE3C9E57F228

Cryptic carp gudgeon

Figures 2 and 3

**Figure 2.**
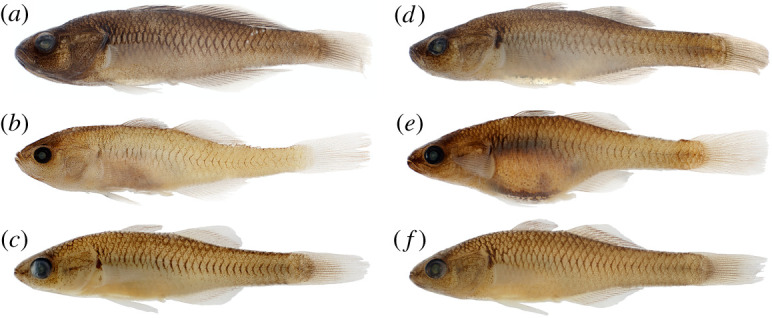
*Hypseleotris acropinna*, holotype, allotype and paratypes. Males are shown on left, females on right. (*a*) NMV A 32246-001, 38.9 mm male, holotype; (*b*) LACM 60028-1, 29.0 mm male, paratype; (*c*) AMS I.47751-008, 31.8 mm male, paratype; (*d*) NMV A 32246-002, 39.8 mm female, allotype; (*e*) LACM 60028-1, 33.7 mm female, paratype; (*f*) AMS I.47751-008, 32.9 mm female, paratype.

**Figure 3.**
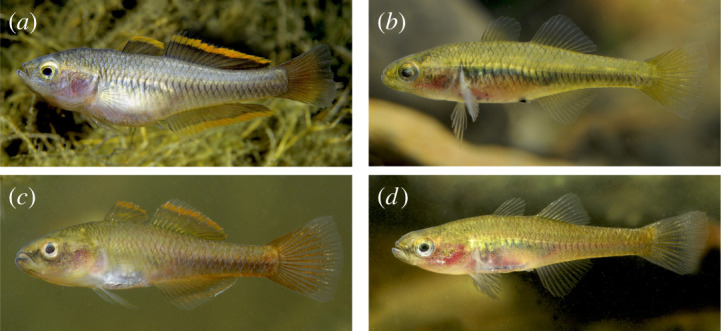
Live photos of *Hypseleotris galii* and *H. acropinna*. (*a*) *H. galii* (male); (*b*) *H. galii* (female); (*c*) *H. acropinna* (male); (*d*) *H. acropinna* (female). The females of *H. galii* have black pigment surrounding the genital papillae that *H. acropinna* females lack. Photos provided by Gunther Schmida.

*Hypseleotris* sp. 3 [10,11,31,32]

*Hypseleotris* sp. 4 (in part) [15,21]

*Hypseleotris* sp. [16]

**Holotype.** NMV A 32246-001, 38.9 mm male (H0525) collected at Little Sideling Creek, a Condamine River tributary north of Barakula, Queensland, Australia (PU15-10), Lat/Long: −26.2452 150.4231

**Allotype.** NMV A 32246-002, 39.8 mm female (H0529), from type locality

**Paratypes.** LACM 60028-1, 29.0 mm male (H0676) & 33.7 mm female (H0679), collected at Castle Creek, a Goulburn River tributary at Euroa, Victoria, Australia (PU15-95), Lat/Long: −36.7839 145.5701

AMS I.47751-008, 31.8 mm male (H1266) & AMS I.47751-009, 32.9 mm female (H1253), collected at Sandy Creek, upper Gregory River, west of Childers, Queensland, Australia (PU17-63), Lat/Long: −25.2120 152.1111

**Diagnosis.** *Hypseleotris acropinna* distinguished among southeastern *Hypseleotris* except *H. galii* and *H. moolooboolaensis* by adult males possessing elongated rays in posterior portions of the second dorsal, anal fins with fin tips extending along length of caudal peduncle. In breeding males, median fins with distal bands of pale orange. Distinguished from *H. galii* by absence of dark pigment around genital papillae in females; *H. galii* females with prominent dark spot ventrally surrounding genital papilla (figure 3). *Hypseleotris acropinna* distinguished from *H. moolooboolaensis* in the Mary River (described below) by *H. acropinna* generally having fewer dorsal spines (VI–VII rather than VII–VIII), fewer dorsal rays (10–11 rather than 11–12), fewer lateral scales (31–33 rather than 33–34). Species very similar, additionally distinguished by their genotypes, geographical ranges.

**Description.** Counts based on individuals of known SNP genotype HA; counts of holotype indicated with asterisk. *Hypseleotris* with first dorsal spines VI–VII* (rarely VIII), second dorsal fin elements I, 10–11* (rarely 12); anal fin elements I, 11–12 (11*; rarely 10 or 13); pectoral fin rays 13–15 (14*); lateral scales 31*–34; head scales 12*–18 on dorsal midline. Head pores absent. Head pointed, with V-shaped snout in profile, unlike rounder profiles of *H. bucephala* and *H. gymnocephala*. Coloration overall pale with dark-edged scales; dark scale margins more intense at lateral midline. Fins clear to dusky except in breeding males exhibiting bright bands on fins as described above. Urogenital papillae in both males, females blunt, with lateral edges of papilla longer than incised centre, yielding w-shaped outline. No dark pigment around the genital papilla of females.

**Figure 4.**
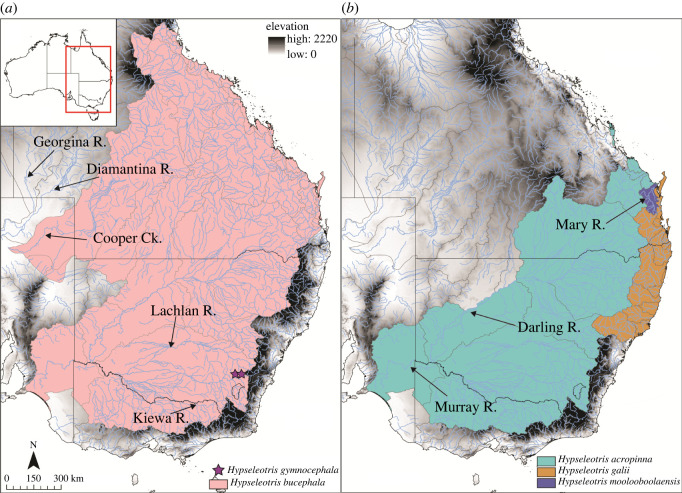
Distribution map for southeastern *Hypseleotris* species. (*a*) Distributions for *H. bucephala* (pink) and *H. gymnocephala* (purple stars); (*b*) Distributions for *H. acropinna* (green), *H. galii* (brown) and *H. moolooboolaensis* (purple). Major river drainages are indicated with arrows.

**Distribution.** Found patchily throughout the MDB as well as eastern coastal streams north of the Mary River to Agnes Waters, with a more northerly disjunct population in Waterpark Creek. Hemiclonal lineages involving this species and both *H. bucephala* and *H. gymnocephala* are known from the MDB, Inman River (South Australian Gulf drainage) and east coast drainages. Introduced populations are also present in several coastal catchments in Victoria and South Australia. A map showing the native distributions of southeastern Australian *Hypseleotris* is given in figure 4.

**Remarks.** The meristic counts agree well with those tabulated in [10], with the exceptions of individuals with dorsal spines ranging from VII to VIII, pectoral fin rays ranging from 14 to 17, and longitudinal scales ranging from 32 to 35 not seen among our genotyped samples. It is possible that the higher counts in [10] are due to inclusion of hemiclones with an *H. acropinna* parent. *Hypseleotris acropinna* is very similar to *H. galii* and new species *H. moolooboolaensis*, and corresponds to the HA allozyme genotype [9,17]. There are three closely related clades recovered in our SNP genotyping analyses: HA in the MDB and coastal populations north of the Mary River, HM from the Mary River, and HG in coastal populations south of the Mary. The MDB and northern coastal populations are *H. acropinna*. The Mary River population is a new species and is described below as *H. moolooboolaensis*. Similar individuals south of the Mary are *H. galii*, and may be distinguished by the presence of black pigmentation around the genital papillae of females that is lacking in the northern and inland populations (figure 3). The only exception to this rule is the *H. acropinna* individuals in the Elliott River, Queensland, which is slightly north (approx. 40 km) of the species boundary at the Mary River. In the Elliott River, females of *H. acropinna* have the black pigmentation surrounding the genital papillae that is characteristic of *H. galii*. In all other respects, including their SNP genotypes, they are concordant with *H. acropinna*, and it is unclear why the characteristic is present in only that population.

**Etymology.** The species name is formed from the Latin roots ‘*acro*’, meaning pointed, and ‘*pinnis*’, meaning fin, describing the elongate, pointed second dorsal and anal fins which help to distinguish them from *H. bucephala*. The term ‘*acropinna*’ is used as a noun in apposition. The common name ‘cryptic’ refers to the extreme difficulty in distinguishing this species from its hemiclone with *H. bucephala*. In addition, it was also long confused with *H. bucephala* in the Murray-Darling Basin, not being recognized until identified by [16,17] despite *H. acropinna* being common and widespread.

***Hypseleotris galii***

Firetail gudgeon

Figures 3 and 5

**Figure 5.**
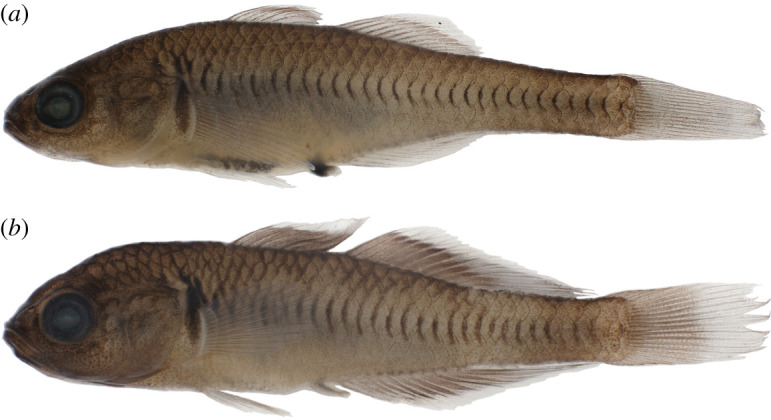
*Hypseleotris galii*, female neotype and male voucher specimen. (*a*) AMS I.49481-001, 28.6 mm female, neotype; (*b*) AMS I.49482-001, 30.3 mm male voucher specimen. This species is distinguished from *H. acropinna* and *H. moolooboolaensis* by the presence of dark pigment on and around the genital papilla in females.

*Hypseleotris galii* [10,11,14,15,21,31]

*Hypseleotris* spp. [33]

**Neotype.** AMS I.49481-001, 28.6 mm female (H2287) collected from Hickeys Creek, a Macleay River tributary near Millbank, New South Wales, Australia (PU18-24), Lat/Long: −30.8645 152.6313

**Male voucher.** AMS I.49482-001, 30.3 mm male (H2266) collected from the Corindi River, west of Corindi Beach, New South Wales, Australia (PU18-22), Lat/Long: −30.0391 153.1197

**Diagnosis.** *Hypseleotris galii* distinguished among southeastern *Hypseleotris* except *H. acropinna* and *H. moolooboolaensis* by adult males possessing elongated rays in posterior portions of second dorsal, anal fins, with fin tips extending along length of caudal peduncle. In breeding males, median fins with distal bands of pale orange, caudal fin dusky red. Distinguished from *H. acropinna* by presence of dark pigment around genital papillae in females; *H. galii* females with prominent dark spot ventrally that surrounds genital papilla (figure 3). *Hypseleotris galii* distinguished from *H. moolooboolaensis* in Mary River (described below) by *H. galii* generally having fewer lateral scales (31–33 rather than 33–34). Species very similar, additionally distinguished by their genotypes and geographical ranges.

**Description.** Counts based on individuals of known SNP genotype HG; counts of holotype indicated with asterisk. *Hypseleotris* with first dorsal spines VII*, second dorsal fin elements I, 11*–12; anal fin elements I, 11*–12; pectoral fin rays 15–16*; lateral scales 31*–33; head scales 12–14 (13*) on dorsal midline. Head pores absent. Head pointed, with V-shaped snout in profile, unlike rounder profiles of *H. bucephala*, *H. gymnocephala*. Coloration overall pale with narrow dark parenthesis-shaped marks between scales at lateral midline. Fins clear to dusky except in breeding males exhibiting bright bands on fins as described above. Urogenital papillae in males, females blunt, with lateral edges of papilla longer than incised centre, yielding w-shaped outline. Prominent dark pigment spot on, around the genital papilla of females.

**Distribution.** Found in eastern coastal streams from south of the Mary River to the Karuah River, with populations further south to the Georges River probably being introduced. Hemiclonal lineages involving this species and *H. bucephala* or *H. gymnocephala* are also known from east coast drainages, although the *H. gymnocephala* hemiclones could be translocations. A map showing the native distributions of southeastern Australian *Hypseleotris* is given in figure 4.

**Remarks.** This is a well-known species, which we redescribe here in order to facilitate its distinction from the other southeastern Australian species and in order to designate a new type specimen. The species was described as *Carassiops galii* [14], from a specimen captured from an artificial pond at the Sydney Botanic Gardens, with dorsal spines VII–VIII; dorsal rays 10–12; anal fin counts I, 11–14; pectoral rays 15; lateral scales 29–31; and vertebrae 30–31 (16 + 14–15). The original source of the fish introduced into the Sydney Botanic Gardens is not known. No figure was provided, no types were designated, and no mention was made in the original description of pigmentation around the genital papilla. A later account of the species [34] described individuals bred from the same Sydney Botanic Gardens population and listed the same meristic counts. A figure was given of a male, showing the elongated tips of the posterior median fins and depicting those fins as proximally dark with a lighter distal margin. No illustration of a female was given so it remains unclear whether or not dark pigment at the genital papilla was present. Due to these uncertainties, we here designate a neotype for *H. galii* and deposit an additional male voucher specimen. Both of these specimens are of confirmed SNP genotype HG.

**Etymology.** Named by J. Douglas Ogilby [14] in honour of his friend, Mr. Albert Gale, who observed the fish in the Sydney Botanic Gardens, suspected it was a new species, and assisted Ogilby with collection of the fish.

***Hypseleotris bucephala* Thacker, Geiger & Unmack sp. nov.**

urn:lsid:zoobank.org:pub:7ECCD2C4-302E-447F-BFCA-AE3C9E57F228

Boofhead carp gudgeon

Figure 6

**Figure 6.**
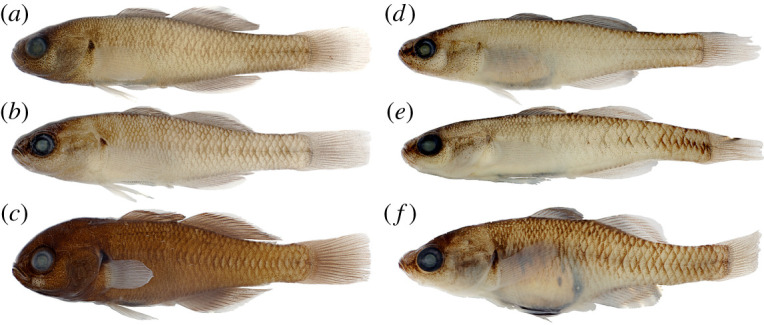
*Hypseleotris bucephala*, holotype, allotype and paratypes. Males are shown on left, females on right. (*a*) NMV A 32244-001, 33.1 mm male, holotype; (*b*) LACM 60026-1, 28.0 mm male, paratype; (*c*) AMS I.47086-002, 29.7 mm male, paratype; (*d*) NMV A 32244-002, 31.8 mm female, allotype; (*e*) LACM 60026-1, 27.7 mm female, paratype; (*f*) AMS I.47086-003, 23.4 mm female, paratype. The specimens in (*c*) and (*f*) are larger adults in breeding condition.

*Hypseleotris* sp. 1 [10] (in part; probably included hemiclones)

*Hypseleotris* sp. 4 [11,15,21,31]

*Hypseleotris* spp. [33]

**Holotype.** NMV A 32244-001, 33.1 mm male (H0713) collected at Lachlan River, below Cargellio Weir, New South Wales, Australia (PU16-08), Lat/Long: −33.2012 146.4523

**Allotype.** NMV A 32244-002, 31.8 mm female (H0727), from type locality

**Paratypes.** LACM 60026-1, 28.0 mm male (H0714) & 27.7 mm female (H0728), from type locality

AMS I.47086-002, 29.7 mm male (H0572) & AMS I.47086-003, 23.4 mm female (H0583), collected in a billabong on the upper Murray River near Tintaldra, New South Wales (PU15-87), Lat/Long: −36.0375 147.9728

**Diagnosis.** *Hypseleotris bucephala* distinguished among southeastern *Hypseleotris* in possessing blunt head, snout rounded in profile, with dorsal scales; lacking pointed median fin tips of *H. acropinna*, *H. galii*, *H. mooloboolaensis*; unique median fin coloration in breeding males with distal half of fin bearing bands of red, black, orange, often with thin edge of white.

**Description.** Counts are based on individuals of known SNP genotype HB; counts of holotype indicated with asterisk. A *Hypseleotris* with first dorsal spines VII*–VIII (rarely VI), second dorsal fin elements I, 10–12*; anal fin elements I, 10–12*; pectoral fin rays 13–15*; lateral scales 30–35*; head scales 0–19 (18*) on dorsal midline. All specimens examined with scales on dorsal head, nape, ranging from well-developed, imbricate to scattered, small, cycloid. In some poorly scaled specimens, no scales present at dorsal midline, but always present elsewhere on head. Head pores absent. Coloration overall tan to brown, with alternating light, dark bands on dorsal half of body. Ventral half paler, with silvery throat, belly. Fins clear to dusky except in breeding males exhibiting bright bands on fins as described above. Urogenital papillae in males, females blunt, with lateral edges of papilla longer than incised centre, yielding w-shaped outline.

**Distribution.** Found throughout the MDB, Bulloo River (Bulloo-Bancannia Basin, BBB), Cooper Creek (LEB), and eastern coastal streams from the Tully River south to around the Brisbane River. Recently confirmed to occur in the upper Einasleigh River (draining into the Gulf of Carpentaria) [35]. Hemiclonal lineages involving this species and *H. acropinna, H. galii, H. moolooboolaensis,* or *H. gymnocephala* are known from the MDB, BBB, LEB and east coast drainages. A map showing the native distributions of southeastern Australian *Hypseleotris* is given in figure 4.

**Remarks.** The meristic counts agree well with those previously tabulated by [10,15]. Counts for fin spines, fin rays and longitudinal scales overlap among *Hypseleotris bucephala* and the other southeastern Australian *Hypseleotris* species. *Hypseleotris bucephala* can be distinguished as one of the two species (the other is *H. gymnocephala*) with a blunted snout, particularly in mature males which develop an expanded hump on the forehead. *Hypseleotris bucephala* is distinguished from *H. gymnocephala* by having scales on the predorsal (nape) region of the head, as opposed to lacking scales on the nape. *Hypseleotris bucephala* corresponds to the HB allozyme genotype [9,17].

**Etymology.** The species name is formed from the Greek roots ‘*bu*’ meaning large, and ‘*kephale*’, Latinized to ‘*cephalus*’ meaning head, describing the blunt profile and enlarged forehead seen particularly in the males. The name takes a feminine ending, as an adjective, in agreement with the gender of the genus name *Hypseleotris*.

***Hypseleotris gymnocephala* Thacker, Geiger & Unmack sp. nov.**

urn:lsid:zoobank.org:pub:7ECCD2C4-302E-447F-BFCA-AE3C9E57F228

Bald carp gudgeon

Figure 7

**Figure 7.**
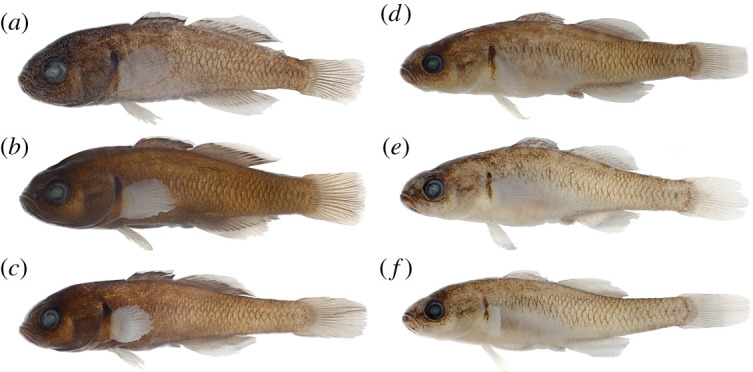
*Hypseleotris gymnocephala*, holotype, allotype and paratypes. Males are shown on left, females on right. (*a*) NMV A 32245-001, 30.5 mm male, holotype; (*b*) LACM 60027-1, 26.8 mm male, paratype; (*c*) AMS I.47540-001, 28.7 mm male, paratype; (*d*) NMV A 32245-002, 31.4 mm female, allotype; (*e*) LACM 60027-1, 29.1 mm female, paratype; (*f*) AMS I.47540-002, 30.1 mm female, paratype.

*Hypseleotris* sp. 1 [10] (hemiclones)

*Hypseleotris* sp. 5 [11,15,21,31] (hemiclones)

*Hypseleotris* spp. [33] (hemiclones)

**Holotype.** NMV A 32245-001, 30.5 mm male (H0372) collected at Meadow Creek, a Lachlan River tributary at Gunning, New South Wales, Australia (PU14-170), Lat/Long: −34.7789 149.2686

**Allotype.** NMV A 32245-002, 31.4 mm female (H0381), from type locality

**Paratypes.** LACM 60027-1, 26.8 mm male (H0373) & 29.1 mm female (H0391), from type locality

AMS I.47540-001, 28.7 mm male (H0377) & AMS I.47540-002, 30.1 mm female (H0387), from type locality

**Diagnosis.** *Hypseleotris gymnocephala* distinguished among southeastern *Hypseleotris* in possessing blunt head with dorsal scales absent as far posteriad as middle of second dorsal fin; lacking pointed median fin tips of *H. acropinna*, *H. galii*, *H. mooloboolaensis*; unique median fin coloration in mature males: distal portion of fin bearing band of pale orange, shading into thin band of white.

**Description.** Counts based on individuals of known SNP genotype HX; counts of holotype indicated with asterisk. *Hypseleotris* with first dorsal spines VII–VIII*, second dorsal fin elements I, 11*–12 (rarely 10); anal fin elements I, 11*–12; pectoral fin rays 15–16* (rarely 17); lateral scales 28–35 (32*); head scales, head pores absent. Coloration overall pale with 6–7 alternating darker patches often visible along length of body. Fins clear to dusky except in breeding males exhibiting bright bands on fins as described above. Urogenital papillae in males, females blunt, with lateral edges of papilla longer than incised centre, yielding w-shaped outline.

**Distribution.** *Hypseleotris gymnocephala* is currently only known from two small creeks in the upper Lachlan River in the MDB. Hemiclonal lineages involving this species and *H. bucephala* are widespread across the MDB as well as Bulloo River (BBB) and Cooper Creek (LEB). Hemiclones with *H. acropinna* are known from the MDB. Some hemiclones have also been recorded from coastal drainages south of the Mary River, including the Brisbane, Clarence and Georges rivers, all within the range of *H. galii*, although these populations could have introduced from elsewhere. A map showing the native distributions of southeastern Australian *Hypseleotris* is given in figure 4.

**Remarks.** The meristic counts agree well with those previously tabulated by [10,15], all of which were based upon hemiclones with an *H. gymnocephala* parent, with the exceptions of dorsal spines ranging up to IX, anal fin elements ranging from I, 10–13, and slightly more longitudinal scales (31–36) in those accounts. Counts for fin spines, fin rays and longitudinal scales overlap among *H. gymnocephala* and the other southeastern Australian *Hypseleotris* species. *Hypseleotris gymnocephala* can be distinguished as one of the two species (the other is *H. bucephala*) with a blunter snout, particularly in breeding males, which also develop an expanded hump on the forehead. By contrast to *H. bucephala,* which may lack scales on the predorsal midline but always has them elsewhere on the nape, *H. gymnocephala* never has scales on the predorsal region. *Hypseleotris gymnocephala* corresponds to the HX allozyme genotype [9,17,22].

**Etymology.** The species name is formed from the Greek roots ‘*gymnos*’ meaning naked, and ‘*kephale*’, Latinized to ‘*cephalus*’ meaning head, describing the scaleless skin of the forehead, nape and dorsal midline anterior to the first dorsal fin characteristic of this species. The name takes a feminine ending, as an adjective, in agreement with the gender of the genus name *Hypseleotris*.

***Hypseleotris moolooboolaensis* Thacker, Geiger & Unmack sp. nov.**

urn:lsid:zoobank.org:pub:7ECCD2C4-302E-447F-BFCA-AE3C9E57F228

Mary carp gudgeon

Figure 8

**Figure 8.**
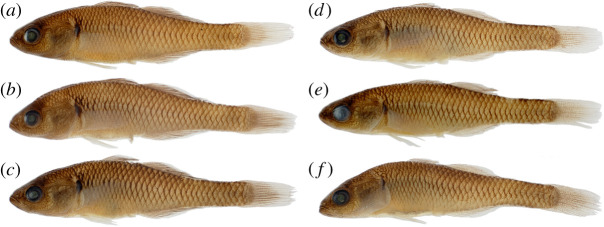
*Hypseleotris moolooboolaensis*, holotype, allotype and paratypes. Males are shown on left, females on right. (*a*) NMV A 32247-001, 33.0 mm male, holotype; (*b*) LACM 60029-1, 34.5 mm male, paratype; (*c*) AMS I.47754-001, 32.8 mm male, paratype; (*d*) NMV A 32247-002, 29.3 mm female, allotype; (*e*) LACM 60029-1, 31.4 mm female, paratype; (*f*) AMS I.47754-002, 31.3 mm female, paratype.

**Holotype.** NMV A 32247-001, 33.0 mm male (H1294) collected at Gutchy Creek, tributary of the Mary River, near Tiaro Queensland, Australia (PU17-66), Lat/Long: −25.7827 152.5247

**Allotype.** NMV A 32247-002, 29.3 mm female (H1298), from type locality

**Paratypes.** LACM 60029-1, 34.5 mm male (H1295) & 31.4 mm female (H1299), from type locality.

AMS I.47754-001, 32.8 mm male (H1296) & AMS I.47754-002, 31.3 mm female (H1303), from type locality

**Diagnosis.** *Hypseleotris moolooboolaensis* distinguished among southeastern *Hypseleotris* except *H. galii*, *H. acropinna* by adult males possessing elongated rays in posterior portions of second dorsal, anal fins with fin tips extending along length of caudal peduncle. Mature males with median fins having distal bands of pale orange. Distinguished from *H. galii* by absence of dark pigment around genital papillae in females; *H. galii* females with prominent dark spot ventrally surrounding genital papilla (figure 3). Distinguished from *H. acropinna* by generally having more lateral scales (33–34 rather than 31–33). Morphologically very similar to *H. acropinna*, species additionally distinguished by their genotypes, geographical ranges.

**Description.** Counts based on individuals of known SNP genotype HM; counts of holotype indicated with asterisk. *Hypseleotris* with first dorsal spines VII–VIII*, second dorsal fin elements I, 10–12*; anal fin elements I, 12*; pectoral fin rays 15*; lateral scales 33–34*; head scales 15–19 (16*) on dorsal midline. Head pores absent. Head pointed, with V-shaped snout, unlike rounder profiles of *H. bucephala*, *H. gymnocephala*. Coloration overall pale with dark-edged scales. Fins clear to dusky except in breeding males exhibiting bright bands on fins as described above. Urogenital papillae in males, females blunt, with lateral edges of papilla longer than incised centre, yielding w-shaped outline. No dark pigment around the genital papilla of females.

**Distribution.** Found in the Mary River and its tributaries. Hemiclonal lineages involving this species and *H. bucephala* are present in the Mary, Maroochy, and Auburn (Burnett) rivers. A map showing the native distributions of southeastern Australian *Hypseleotris* is given in figure 4.

**Remarks.** The meristic counts agree with the higher range of counts previously tabulated for *H. acropinna* [10]. *Hypseleotris moolooboolaensis* is very similar to *H. galii* and *H. acropinna*, and the three species form a clade as discussed above under the description of *H. acropinna*. The morphological differences between *H. acropinna* and *H. moolooboolaensis* are slight, but due to the additional genetic and geographic distinctions, we elect to formally describe the species.

**Etymology.** The species name is derived from the Aboriginal name for the Mary River, Moolooboola. The suffix *-ensis* indicates reference to a geographic locality.

## 4. Unisexual hybrid hemiclonal lineages

**Figure 9.**
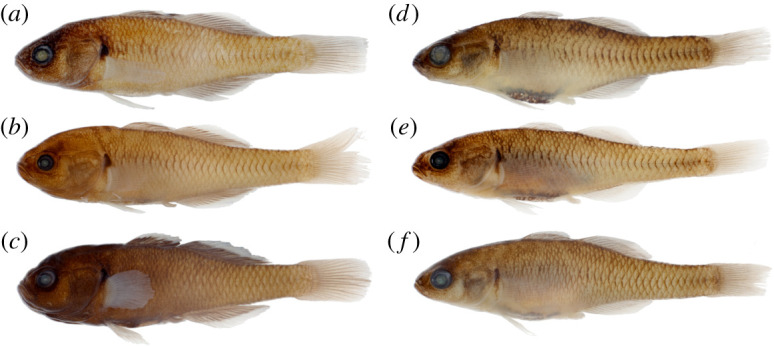
Hemiclones among southeastern *Hypseleotris* species. (*a*) *H. acropinna* × *H. bucephala* 31.9 mm male hemiclone. (*b*) *H. acropinna* × *H. gymnocephala* 33.5 mm male hemiclone. (*c*) *H. bucephala* × *H. gymnocephala* 30.5 mm male hemiclone. (*d*) *H. acropinna* × *H. bucephala* 36.9 mm female hemiclone. (*e*) *H. acropinna* × *H. gymnocephala* 34.6 mm female hemiclone. (*f*) *H. bucephala* × *H. gymnocephala* 31.8 mm female hemiclone.

All of the sympatric *Hypseleotris* species in southeastern Australia can hybridize to form stable, hemiclonal lineages except for *H. klunzingeri* and *H. compressa*. Most of the possible hybrid combinations are known. These hemiclonal lineages are present at most locations and are often abundant. *Hypseleotris bucephala* and *H. gymnocephala* have extensively formed hybrid hemiclonal lineages that are found throughout the MDB, Cooper Creek and the Bulloo River. The closely related species *H. acropinna*, *H. moolooboolaensis* and *H. galii* do not hybridize with one another as far as we know, as each species is allopatric except for a couple of unusual occurrences of *H. moolooboolaensis* in the Maroochy and Auburn (Burnett) rivers (that require further investigation); however, each will form hybrid hemiclones with either *H. bucephala* or *H. gymnocephala* (the exception is the *H. gymnocephala* /*H. moolooboolaensis* pairing, which is not likely since those species do not co-occur). In the cases of *H. acropinna* and *H. bucephala*, at least one of the parental sexual lineages coexist with their hemiclones. For *H. gymnocephala*, the pure parental sexual lineage is very rare, known only from two creeks that flow into the upper Lachlan River in New South Wales, thus any of its hemiclones do not co-occur with it today. In all other localities, the individuals previously identified as Lake's carp gudgeon are hemiclones. Hemiclones may be variable and difficult to distinguish from their parent taxa. Figure 9 shows males and females of the most common hemiclonal lineages: those involving *H. acropinna*, *H. bucephala* and *H. gymnocephala*.

*Hypseleotris acropinna* forms hybridogenic lineages with both *H. bucephala* (figure 9*a*,*d*) and *H. gymnocephala* (figures 9*b*,*e*). The *H. acropinna*-*bucephala* hemiclones are equivalent to the previously identified allozyme genotype HAxHB, and have the following meristic counts: dorsal spines VI–VII; second dorsal elements I, 11–12 (rarely 10); anal fin elements I, 11–12 (rarely 13); pectoral fin rays 14–15; lateral scales 31–34; head scales 10–16 at dorsal midline; head pores absent. These counts overlap broadly with both parental taxa. The sex ratio in these hemiclones is highly biased towards males; 98% of fish sampled in the Goulburn River system (Victoria) were males [9], and we confirm that male bias exists in our samples from across the MDB. The overall morphology of the *H. acropinna*-*bucephala* hemiclones is intermediate between the parental taxa with regard to head and snout shape, and the length/shapes of the median fins. However, because these fish do not generally have the rounded head typical of *H. bucephala*, they are generally identified as *H. acropinna*. At present precise identification as the pure *H. acropinna* species or a hemiclone is not easily possible without genotyping.

**Figure 10.**
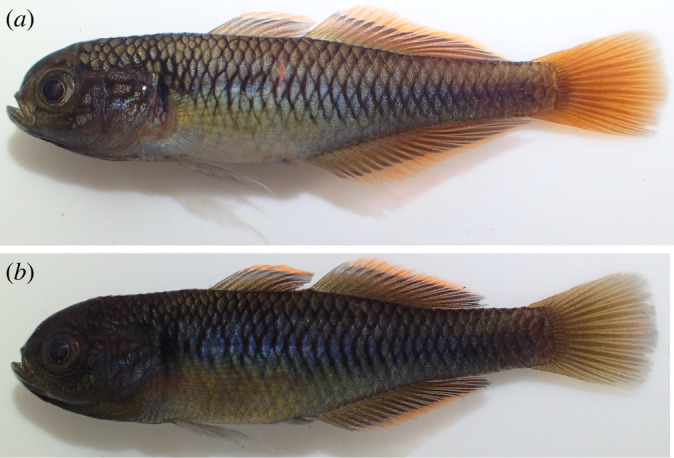
Comparison between mature male *Hypseleotris galii* and *H. bucephala* × *H. galii* hemiclone. (*a*) Mature male *H. galii*. (*b*) Mature male *H. bucephala* × *H. galii* hemiclone. These fish were both collected at Ewen Maddock Dam, Addington Creek, southeast Queensland (tributary of the Mooloolah River, Lat/Long: −26.7907 152.9868). Photo by Karl Moy.

Hemiclones between *Hypseleotris bucephala* and *H. galii* have the following meristic counts: dorsal spines VIII; second dorsal elements I, 12; anal fin elements I, 12; pectoral fin rays 15; lateral scales 33; head scales 16 at dorsal midline; head pores absent. The similar hemiclone pairing between *H. bucephala* and *H. moolooboolaensis* exhibits counts of: dorsal spines VIII; second dorsal elements I, 11–12; anal fin elements I, 12; pectoral fin rays 15; lateral scales 33–34; head scales 16–18 at dorsal midline; head pores absent. These counts are within the ranges of counts for the more common *H. acropinna* × *H. bucephala* hemiclones, except that the *H. bucephala* × *H. galii* and *H. bucephala* × *H. moolooboolaensis* hemiclones have one more dorsal spine. The overall appearance of the individuals is likewise very similar, and they are generally identified as the non-*H. bucephala* parent. Figure 10 shows mature males of *H. galii* and an *H. bucephala* × H. *galii* hemiclone; they are very similar, but the *H. bucephala* × *H. galii* hemiclone is slightly more stocky, has slightly shorter dorsal and anal fins with more orange coloration, and a dusky tail (rather than red).

Hemiclones between *Hypseleotris acropinna* and *H. gymnocephala* are more distinctive, generally resembling more slender *H. gymnocephala*. These hemiclones are equivalent to the previously identified allozyme genotype HA × HX, and have the following meristic counts: dorsal spines VII–VIII; second dorsal elements I, 10–11 (rarely 12); anal fin elements I, 10–12; pectoral fin rays 14–15; lateral scales 31–35; head scales sometimes absent, or if present, 10–16 at dorsal midline; head pores absent. The counts overlap broadly with both parental taxa, and the primary diagnostic character of *H. gymnocephala*, the absence of head and nape scales, is variably expressed. These individuals are also highly sex-biased, but in this case in favour of females, confirming the 96% predominance of females collected in the Goulburn River [9]. These fish may be mis-identified as *H. acropinna* if the head is scaled and more pointed.

Finally, hemiclones between *Hypseleotris bucephala* and *H. gymnocephala* correspond to the previously identified allozyme genotype HB × HX, and have the following meristic counts: dorsal spines VII–IX; second dorsal elements I, 10–12; anal fin elements I, 11–12; pectoral fin rays 14–16; lateral scales 33–37; head scales and head pores absent. These counts overlap with those of their parental taxa in most respects, except that the dorsal spine count may be as high as IX, and the lateral scales may be as high as 37; the body may be slightly more elongate. The sex ratio in these hemiclones is not skewed, with roughly equal numbers of females and males [9]. This hemiclonal combination is common across the MDB, Bulloo River (BBB) and Cooper Creek (LEB) and is relatively easy to identify morphologically as scales are absent from the head and part of the upper dorsal surface, usually to the level of the middle of the first dorsal fin, although the extent of scalelessness varies. They also lack scales on their belly up to the level of the middle or top of their pectoral fin. This hybrid combination also exhibits the blunt heads typical of both parental lineages (figure 9*c*,*f*). They are most likely to be confused with *H. acropinna* × *H. gymnocephala* hemiclones which typically have a smaller scaleless patch on their head. They are unlikely to be confused with *H. gymnocephala* due to the extremely limited distribution of the pure parental lineage, thus, any bald-headed *Hypseleotris* found are almost certainly hemiclones.

## 5. Discussion

A primary challenge of taxonomy is reconciling the names assigned to taxa with the lineages that actually exist in nature. Species concepts are many and have engendered a long history of debate (reviewed in [36]) which we will not summarize here. Fundamentally, a species is a separately evolving metapopulation lineage (general lineage species concept [37]) which may be identified and delimited based on any number of genetic, morphological, ecological or behavioural characteristics. Practical difficulties arise from the biological reality that diverging lineages accumulate those characteristics separately and at different times. Another difficulty is that a range of ecologies, reproductive systems, and lineage cohesion mechanisms exists among species in nature so identifying divergent characteristics by which to distinguish species can be different in each individual case. Ideally, several lines of evidence will support the hypothesis that separate lineages (species) are present among a population of individuals [37,38].

Zoologists typically evaluate species boundaries with reference to sexually reproducing, bisexual lineages, which is why reproductive incompatibility, confirmed or inferred, is such a common species recognition criterion. Generally, animal species are also assumed to have arisen by divergence from other lineages rather than by reticulation among lineages. However, some organisms reproduce unisexually (clonally or hemiclonally) and scientific names may also be applied to those lineages [6]. Unisexual lineages in fishes are generally dependent on their parental taxa for reproduction; the gynogenesis is sperm-dependent in that all female species need sperm to activate the eggs, although there is no (or little) genetic contribution from the male [4]. Hybridogenesis in these systems is often ongoing, with matings resulting in new hemiclones constantly being generated from parental taxa [6,39,40]. Among *Hypseleotris*, the situation may be even more complicated in that it has been proposed that some hemiclones (*H. bucephala × H. gymnocephala*) may be generated from matings of two hemiclonal taxa (*H. acropinna* × *H. bucephala* and *H. acropinna × H. gymnocephala*) in addition to direct mating with a sexual species [9].

The naming of unisexual lineages is rare enough that there is not a commonly accepted practice. Names of unisexual taxa may be expressed as Genus species A × Genus species B, or as Genus species A-species B [4,41]. In some cases, the unisexual species has been given its own formal name, as with *Menidia clarkhubbsi*, a silverside from Texas that is all-female and a sperm parasite of its paternal parent taxon [42]. It is likely that at least one of the parental species is still extant based on the mtDNA and allozyme profiles of the hemiclones [43]. The unisexual minnow *Squalius alburnoides* arose from hybridization involving a *S. pyrenaicus* female and an unknown, extinct male parent [39]. This hybrid origin was presumably not known when the species was described [44]. Among *Cnemidophorus* lizards, some unisexual lineages have been given standard scientific names (Genus species or Genus species complex), even when their hybrid origin is known, a practice validated by the International Code of Zoological Nomenclature [6,45].

In hybridogenic amphibians, the male genotype is sometimes incorporated into the fertilized egg, resulting in polyploidy if the maternal diploid genome is maintained or diploidy if half of the maternal genetic contribution is discarded. This strategy is known as kleptogenesis, and the resulting lineage is a klepton. This pattern has been identified in *Ambystoma* salamanders [1] and *Pelophylax* water frogs [46]. In cases where kleptogenesis is documented, the species may be designated with a kl. between the genus and species name: Genus kl. species.

In the case of southeastern Australian *Hypseleotris* (excluding *H. compressa*), five sexual species are known to form hybridogens and can be identified morphologically and genetically; four of those species are formally described here. Historical hybridization resulting in the formation of hemiclonal lineages is common among those five species and the resulting lineages, while identifiable genetically, are not 100% distinguishable from their parental taxa. Because several species are involved in the generation of hemiclones, it would be unwieldy to refer to this system as the *H. acropinna*/*H. bucephala*/*H. galii*/*H. gymnocephala/H. moolooboolaensis* complex. The precise mechanisms behind *Hypseleotris* hybridogenesis are still unclear but there is no evidence for kleptogenesis or polyploidy among the hemiclones, so we would not use the kl. designation in a species name. Although it would be possible to assign separate scientific names to the unisexual *Hypseleotris* lineages, we have elected not to do so. For each hemiclonal lineage we recommend using the combined scientific names from both parental species and/or their associated genotype codes, as per previous published work, for instance, *H. acropinna* × *bucephala* (HA × HB), *H. acropinna × gymnocephala* (HA × HX), *H. bucephala × gymnocephala* (HB* ×* HX), *H. bucephala* × *H. galii* (HB × HG), or *H. bucephala × H. moolooboolaensis* (HB × HM). To avoid confusion with previous literature we recommend discontinuing previous common names used in this group (Lake's, Midgley's and Murray-Darling carp gudgeon) as they are now known to refer to multiple lineages. While many morphological characters of the parental species and hemiclones broadly overlap, hemiclonal combinations involving *H. gymnocephala* can be distinguished by their differing degree of baldness and to a lesser extent by their sex ratio and their current known distribution. Hemiclones involving *H. acropinna*, *H. galii*, or *H. moolooboolaensis* with *H. bucephala* are difficult to distinguish morphologically. Although subtle differences in body depth, fin length (in males) and coloration (in males) exist, it is not yet known if those differences are consistent among the hemiclone pairings.

## 6. Conclusion

We use analysis of morphology correlated with SNP genotyping to untangle a difficult taxonomic problem: identification and description of pure parental taxa in a hemiclonal hybrid species complex of Australian freshwater fishes. After genotyping and examination of *Hypseleotris* individuals from six lineages in central and eastern Australia, we clarify the identity of five taxa that participate in the formation of hemiclonal hybrids and assign four of them new species names: *H. acropinna*, *H. bucephala*, *H. gymnocephala* and *H. moolooboolaensis*. We further designate a neotype and provide an updated description for the fifth hemiclone-forming species, *H. galii*. The discernment and identification of parental species and hemiclone hybrids in this complex is crucial to documenting and preserving these native Australian fishes and their habitats. We provide morphological counts, photographs and descriptions to facilitate these identifications, allow for better informed conservation planning and also to aid in unravelling the evolutionary history of this unique group.

## Data Availability

The datasets supporting this article have been uploaded as part of the electronic supplementary material [47].
